# Social Structure and Genetic Distance Mediate Nestmate Recognition and Aggressiveness in the Facultative Polygynous Ant *Pheidole pallidula*

**DOI:** 10.1371/journal.pone.0156440

**Published:** 2016-05-31

**Authors:** Denis Fournier, Jean-Christophe de Biseau, Sophie De Laet, Alain Lenoir, Luc Passera, Serge Aron

**Affiliations:** 1 Evolutionary Biology & Ecology, CP 160/12, Université libre de Bruxelles, Avenue F.D. Roosevelt 50, 1050, Brussels, Belgium; 2 IRBI, Institut de Recherche sur la Biologie de l’Insecte, UMR CNRS 7261, Université François Rabelais, Parc Grandmont, 37200, Tours, France; 3 Centre de Recherches sur la Cognition Animale—UMR 5169, Université Paul-Sabatier, 31062, Toulouse, France; Universidade de São Paulo, Faculdade de Filosofia Ciências e Letras de Ribeirão Preto, BRAZIL

## Abstract

In social insects, the evolutionary stability of cooperation depends on the privileged relationships between individuals of the social group, which is facilitated by the recognition of relatives. Nestmate recognition is based on genetically determined cues and/or environmentally derived chemical components present on the cuticle of individuals. Here, we studied nestmate recognition in the ant *Pheidole pallidula*, a species where both single-queen (monogyne) and multiple-queen (polygyne) colonies co-occur in the same population. We combined geographical, genetic and chemical analyses to disentangle the factors influencing the level of intraspecific aggressiveness. We show that encounters between workers from neighbouring colonies (*i*.*e*., nests less than 5 m away) are on average less aggressive than those between workers from more distant colonies. Aggressive behaviour is associated with the level of genetic difference: workers from monogyne colonies are more aggressive than workers from polygyne colonies, and the intensity of aggressiveness is positively associated with the genetic distance between colonies. Since the genetic distance is correlated with the spatial distance between pairs of colonies, the lower level of aggression toward neighbours may result from their higher relatedness. In contrast, the analysis of overall cuticular hydrocarbon profiles shows that aggressive behaviour is associated neither with the chemical diversity of colonies, nor with the chemical distances between them. When considering methyl-branched alkanes only, however, chemical distances differed between monogyne and polygyne colonies and were significantly associated with aggressiveness. Altogether, these results show that the social structure of colonies and the genetic distances between colonies are two major factors influencing the intensity of agonistic behaviours in the ant *P*. *pallidula*.

## Introduction

Kin selection, *i*.*e*. the preferential treatment of genetic relatives [1, 2], has been extremely successful in explaining the evolution of altruistic behaviours, ranging from cells giving up their own survival to help other cells’ dispersal in slime moulds, to worker sterility or suicidal defence in social insect colonies [3–7]. However, kin selection also applies to competition when relatives compete less with each other [8]. In this situation, individuals increase their direct fitness by not spending resources on competition and their indirect fitness by not reducing the fitness of kin. Recognition of relatives from strangers therefore greatly facilitates kin selection, since it allows differential treatment of conspecifics according to their relatedness thereby reducing the costs of positive interactions [9, 10].

Social insects, especially ants, are valuable biological models for investigating the recognition systems that operate in a social context, for at least two reasons. First, recognition and discrimination of nestmates from non-colony members are well-documented traits of ants. Workers typically defend their nest and territory against foreign conspecifics, which helps maintain the genetic integrity of the colony and the safeguarding of resources from competitors, robbers or social parasites [11–14]. Nestmate recognition is based on the perception of non-volatile olfactory cues that are contained in the lipid layer covering the insect cuticle. Their chemical composition can be affected by the genetic makeup of the colony [15–17], environmental factors (diet, habitat, nest material) [18, 19] or both [20]. The colony-specific odour results from the homogenization of cues among workers through trophallaxis and allogrooming [15, 21–25] and it is thought to be used as a recognition template against which to compare labels of other individuals [12, 26–29]. Empirical evidence has shown the central role of cuticular hydrocarbons (CHCs) as the main cues in intraspecific discrimination in ant species [13, 14, 27, 30, 31]. Aggression probabilities and the intensity of agonistic behaviours correlate with dissimilarity in cuticular hydrocarbon profiles and/or the chemical distance between colonies [19, 32–34]. In addition, aggressive behaviour may depend on the variation in the CHC profile within colonies [26, 35–37]. Workers from colonies with greater odour-cue diversity (*e*.*g*., due to the co-existence of several nestmate queens) are expected to be more tolerant toward intruders than workers from colonies with less diversity, because their acceptance threshold is likely to be higher [26, 38]. Second, the kin structure of colonies may vary greatly both between and within species, allowing exploration of the level of aggression as a function of genetic relatedness between individuals. Ants indeed show large variations in their social organization. For example, colonies of a given species may differ in the number of reproductive queens, which may vary from one (monogyny) to several dozen (polygyny) in the same population [39–42]. Although polygyny reduces intra-colony relatedness, hence worker fitness benefits, adoption of new reproductive females may result from nest site limitation or high risks associated with independent colony founding. It may also be selected for if the occurrence of multiple queens enhances colony productivity, longevity or resistance to pathogens due to increased genetic diversity [35, 43–53]. Social polymorphism brings an additional level of complexity to the study of nestmate recognition, because it is often associated with changes in dispersal behaviour and colony foundation [43, 44, 46]. Usually, monogyny involves long-range nuptial flights and independent colony foundation (without the help of workers), whereas colony reproduction under polygyny proceeds by budding, a process whereby freshly mated queens leave their natal nest with a worker force to found new colonies nearby. Budding typically results in population viscosity in which aggressiveness of workers from neighbouring colonies can be affected by both their common origin (genetic similarity) and/or their spatial proximity (environmental cues similarity).

When nestmate recognition involves genetically based recognition cues, two general predictions can be made. Firstly, workers are expected to respond more aggressively toward genetically divergent individuals, because they are chemically less similar [54, 55]. Secondly, variation in the colony kin structure is expected to be associated with differences in the level of aggression shown toward intruders. This is because a greater genetic diversity within colonies, due to the occurrence of multiple queens, is likely to result in a broader blend of cues characteristic of the colony odour. This may decrease the accuracy of nestmate recognition and, ultimately, compromise the workers’ ability to discriminate against non-nestmate conspecifics [35–37]. In line with this, workers from polygyne colonies have been shown, on average, to be more tolerant of and less aggressive toward non-nestmates than workers from monogyne colonies in some ants [43, 56–59]. However, workers’ aggressive behaviour does not always co-vary with genetic divergence and/or colony kin structure [23, 60–63]. This may result from colony-level selection on genetically diverse colonies to reduce within-colony aggression, if it reduces overall productivity, for example by producing a uniform colony recognition odour.

We report results from a multifactorial analysis investigating nestmate recognition in the socially polymorphic ant *Pheidole pallidula*. In this species, both single-queen and multiple-queen colonies coexist in close proximity in the same populations. Kinship among co-breeding queens varies across colonies, from full-sisters to unrelated queens [41, 64–66]. We combined data from behavioural assays, genetic markers and cuticular hydrocarbon profiles to explore the association between genetic distance, chemical signature and nestmate recognition. First, we explored the effect of the social origin of the workers (monogyne or polygyne) on the level of aggressiveness. We examined whether workers originating from polygyne colonies are more tolerant toward foreign conspecifics than workers from monogyne colonies. We also tested for an association between kinship and nestmate recognition. Second, we tested the role of kinship on nestmate recognition by investigating whether the degree of genetic divergence between colonies predicts the level of aggressiveness. Third, we analysed the pattern of variation of worker cuticular hydrocarbons in relation to the social structure, and tested for a possible relationship between the genetic and chemical distances. To determine the potential role of these compounds as labels for nestmate recognition, we correlated levels of intraspecific aggression between colony pairs with the similarity of their cuticular lipid profiles. Finally, we tested for an association between the spatial distance between colonies, on the one hand, and workers’ aggressive behaviour, genetic distance and chemical distance between colonies, on the other.

## Methods

### Sampling and nest distribution

**Fig 1:**
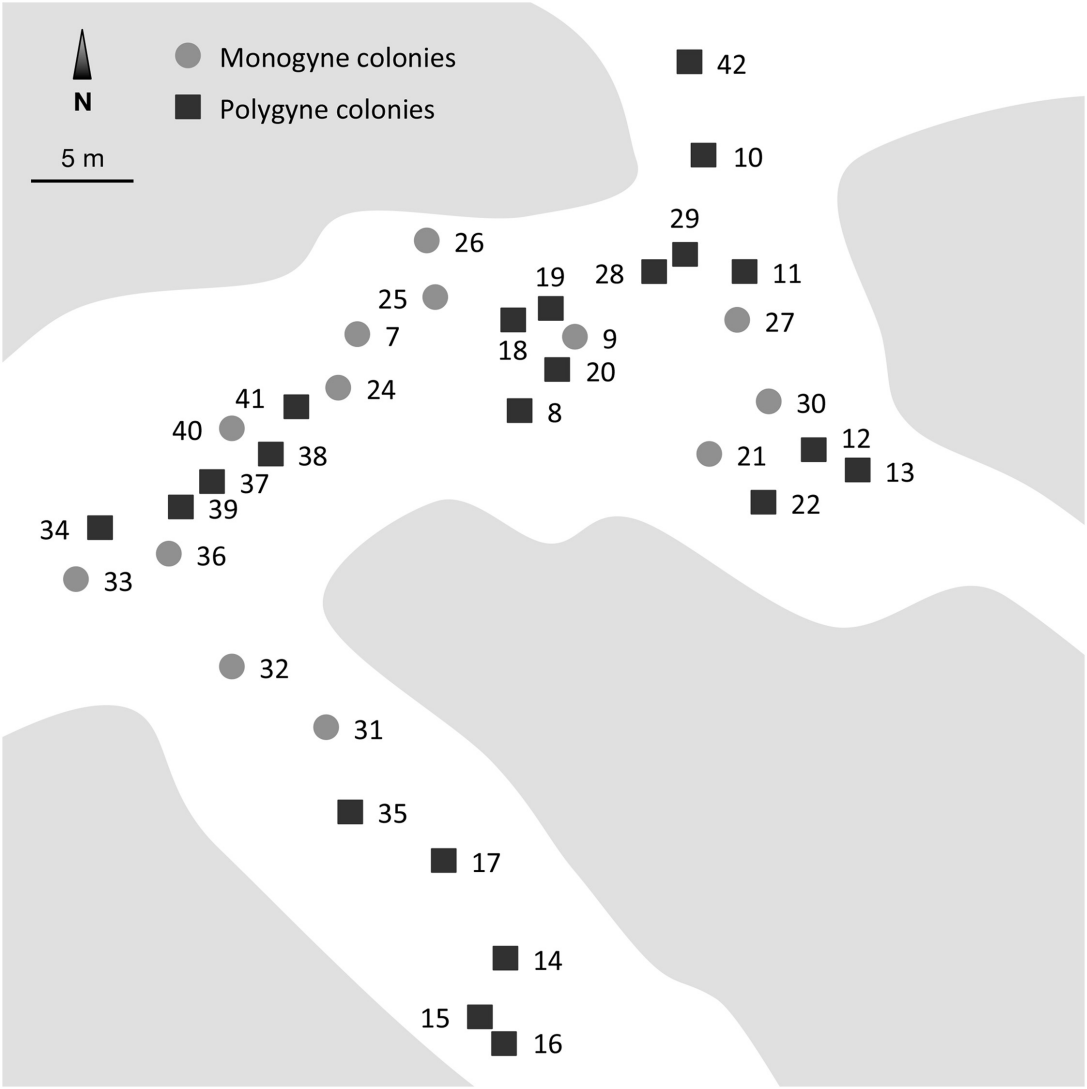
Map of the study area of *Pheidole pallidula* at Bruniquel (Tarn-et-Garonne, France; N44.05011 E1.65621). Filled circles: monogynous colonies (*n* = 13). Filled squares: polygynous colonies (*n* = 22). Grey areas represent sparse vegetation composed mainly of grasses, as well as junipers (*Juniperus communis*) and downy oaks (*Quercus pubescens*) (S1 Table).

The study was conducted in April 2010 in a facultatively polygynous population of *Pheidole pallidula* located in Bruniquel (Tarn-et-Garonne, France; N44.05011 E1.65621; no specific permissions were required for this location, and the field studies did not involve endangered or protected species; [41]). Previous studies in this population showed no sign of genetic differentiation between monogyne and polygyne colonies, indicative of gene flow between both social forms [41, 65]. Moreover, colonies are genetically differentiated and form a population exhibiting low but significant isolation-by-distance, suggesting that some colonies originate through budding [41]. The site consists of relatively sparse vegetation composed mainly of grasses and a few junipers *Juniperus communis* and young downy oaks *Quercus pubescens* [67]. We mapped all nests in a 50 m x 50 m area to determine their spatial distribution, and collected a sample of workers from each nest (Fig 1). The distances between adjacent collection points ranged from 0.77 m to 41.24 m (mean ± SD = 17.58 ± 8.98 m, *N* = 36). A chi-square test was used to verify if colonies’ distribution differed from a simulated random spatial Poisson distribution. We computed a coefficient of dispersion (CD) equal to the observed variance divided by the observed mean to characterise the deviation of colonies distribution (*CD* ≈ 1 in an evenly colonies distribution, > 1 in clumped samples, and < 1 in cases of repulsion; [68]).

The Mediterranean ant *Pheidole pallidula* is characterized by a dimorphism of the sterile caste (with soldiers and minor workers), but because most of the sterile individuals exploring the soil surface and collecting food belong to the minor caste, we used only minor workers in our analyses [67, 69]. We collected a large sample of workers from each colony. A sub-sample of 50 minor workers from each nest was immediately used for chemical extraction (see below). From these, twelve individuals per nest were then randomly chosen and stored in ethanol for subsequent genetic analyses. The remaining workers were kept alive for behavioural assays.

### Behavioural assays

Dyadic aggression tests were performed directly after collection. Assays were therefore conducted blind as the observer who recorded worker aggression levels could not know the social structure of the colonies. Intraspecific aggressiveness was quantified between pairs of minor workers according to previously described protocols [69, 70]. Behavioural interactions between the two individuals were observed and scored over 5 min according to escalating aggression on a scale from 0 to 4: 0—ignore, 1—short antennations (< 2 sec.), 2—prolonged antennations, 3—aggression (lunging, attempts at biting, pulling and mandible grasping) and 4—prolonged aggression and fighting. Categories 0 to 2 were regarded as non-aggressive while 3 and 4 were aggressive. Five trials were conducted for each pair of colonies but each worker was used only once. We averaged the highest score obtained in each of the five replicates to yield a mean aggression level. A posteriori, experimental assays consisted of confrontations between workers from monogyne colonies (M x M), from polygyne colonies (P x P) or from monogyne and polygyne colonies (M x P), while control experiments consisted of confrontations between nestmates from monogyne or polygyne colonies. We also set up encounters between majors from different nests to confirm that workers from both castes did not behave differently when confronted with non-colony members.

### Microsatellite analysis

Colony genetic diversity and population genetic structure were examined by genotyping four statistically independent, highly polymorphic DNA-microsatellite loci (*Ppal-03*, *Ppal-12*, *Ppal-84* and *Ppal-01T*; [41]). DNA was isolated through phenol/chloroform extractions and ethanol precipitation by following standard protocols [41, 71]. Multiplex PCRs were performed in a 10 μl mix containing 2x Qiagen Multiplex PCR Master Mix (Qiagen Inc.), 2 μM of each primer and 1 μl of genomic DNA (about 10 ng of DNA). PCR were carried out using a PTC-200 Peltier thermal cycler (MJ Research Inc.). After an initial denaturing step of 15 min at 95°C, the PCR consisted of 30 cycles of 30 s at 94°C, 90 s at 61°C, and 90 s at 72°C, followed by a final extension step of 30 min at 72°C. Microsatellite loci were analysed using an ABI 3730 automated sequencer (Applied Biosystems, Foster City, CA, USA); the lengths of PCR products were determined using Genemapper software (Applied Biosystems) and used to construct a multilocus genotype for each individual.

We first examined whether the samples of workers collected at different locations belonged to the same colony or not. We performed genotypic tests of differentiation between all pairs of collection points by means of the log-likelihood (*G*) based exact test [72] as implemented in the program Genepop on the Web [73, 74] (http://genepop.curtin.edu.au/). The overall significance was determined for each comparison using Fisher’s combined probability test. A Bonferroni correction based on the number of loci used was applied to account for multiple comparisons (*α* = 0.0125) [75].

The social structure (monogyne or polygyne) was determined by analysing the genotypes present within each colony. Genetic analyses of mother–offspring combinations and of the sperm stored in the spermatheca of queens has previously shown that queens of *P*. *pallidula* are strictly monandrous (effective paternity number per colony *Me* = 1 [41]); therefore, each queen was assumed to contribute two alleles to the allelic pool, and males one allele. The minimum number of queens per colony is therefore inferred from the number of alleles detected among twelve workers at each locus: 3 or fewer alleles = 1 queen, 4–6 alleles = 2 queens, etc. [60]. Genotypes were visually inspected, and the most parsimonious sibship reconstruction was kept for each colony. Since queens of *P*. *pallidula* are obligatorily singly mated, reconstruction of families in monogynous colonies was straightforward. The minimum number of matrilines per colony was confirmed by using the maximum-likelihood methods implemented in the program Colony 1.1 [76], assuming multiple singly-mated queen per nest. Five replicate runs yielded similar results.

Genetic variation was assessed through the mean gene diversity *He* [77] over loci within colonies using Fstat 2.9.3.2 [78]. The genetic distances between pairs of colonies were estimated by the pairwise *F*_*ST*_ values [79], as implemented in the computer program GenAlEx 6 [80].

### Chemical analyses

Groups of 50 workers per colony were killed at -20°C and deposited in 1 ml of cyclohexane for five minutes. The solvent was then evaporated until 10 μl remained. Three μl were injected into a FID gas-chromatograph (VGM250Q system, Perkin-Elmer, Norwalk, CT, USA) equipped with a split/splitless injector and flame ionization detector that used a DB-5 fused silica capillary column (30 m by 0.25 mm; film thickness 0.10 μm). The temperature was kept at 150°C during the initial splitless 2 min, raised from 150°C to 300°C at 5°C/min, and held at 300°C for the last 10 min. Helium was used as carrier gas, with a constant flow rate of 2.0 ml/min. The non-volatile cuticular lipids were identified with the same GC coupled to a Perkin-Elmer MS operating at 70 EV. Standards made up of a ladder of linear alkanes (C_20_, C_22_, C_24_… to C_40_) dissolved in cyclohexane were injected between every few samples to provide a reference set of retention times. We also injected one cuticular extract into a high temperature column (DB-5HT) at 370°C to check if some hydrocarbons with high molecular weights appeared [27]. As no more hydrocarbons were detected, we subsequently used the normal DB-5 column. The areas of peaks were measured by peak integration with a Perkin Elmer Turbo-Chrome Workstation.

Chemical diversity of cuticular hydrocarbon (CHC) profiles were assessed using the Gini-Simpson index *H*_*GS*_ = 1−Σ*p*_i_^2^ (where *p*_i_ is the relative proportion of the *i*-th peak over all chemical peaks identified as cuticular lipids, and *i* ranges from 1 to the total number of peaks present) and the chemical richness *Cr*, *i*.*e*. the number of compounds in an individual sample. Chemical similarity of CHC profiles between colonies was estimated from the relative proportion of each compound using the Euclidian distance *E* [81]. Principal component analysis (PCA) was used to transform the variables into uncorrelated components, and discriminant analysis (DA) was carried out on principal components to visualize the variations among colonies (by maximizing the between-colony variation and minimizing intra-colony variation). Because the percentage abundance of each compound depends on the relative abundance of other compounds present, peak areas were standardized following Aitchison’s formula: *Z*_*i*,*j*_ = ln[*Y*_*i*,*j*_/g(*Y*_*j*_)], where *Z*_*i*,*j*_ is the standardized peak area *i* for colony *j*, *Y*_*i*,*j*_ is the peak area *i* for colony *j*, and g(*Y*_*j*_) is the geometric mean of all peaks for colony *j* [82]. To apply the transformation formula on profiles with non-detectable components, the constant 3 (one-tenth of the smallest area measured) was added to all peak areas. Two-group statistical comparisons for each compound were performed using Mann-Whitney *U*-tests with the significance level set at 0.0008 (*i*.*e*. 0.05 divided by the number of peaks).

### Statistical analyses

Multiple tests were performed with each focal colony. To avoid pseudoreplication, we treated each colony as a statistical unit. We then used between-nest mean values per colony and mean comparison tests for independent samples to compare monogyne and polygyne colonies [83]. Because colonies are not independent from each other, pairwise relationships were measured through correlation matrices, with the significance of the association tested using Mantel tests in GenAlEx with 999 permutations. Deviations of the variables from normality were tested using the Shapiro-Wilk test. When logarithmic or angular transformations did not suffice to normalize the data, we used nonparametric statistics [68]. Statistical tests were carried out with the computer program SPSS 20.0 (SPSS Inc., 1989–2011). Post hoc power analyses for *p*-values between 0.05 and 0.20 were conducted to estimate the probability of a Type II error (not rejecting a false null hypothesis) using the computer program G*Power [84, 85] and the package *biotools* (function *mantelPower*, [86]) in *R* version 3.1.3 [87].

## Results

Our genetic analyses confirmed that the four DNA-microsatellite loci under study were unlinked and segregating independently, and that allelic frequencies were in Hardy-Weinberg equilibrium. The DNA-microsatellite loci *Ppal-03*, *Ppal-12*, *Ppal-84* and *Ppal-01T* had 17, 13, 11 and 14 alleles, respectively, and a level of observed heterozygosity ranging from 0.809 to 0.846. Two collection points located 1.09 m apart were not significantly differentiated (they probably represented two samplings of the same colony); one of these was therefore discarded at random from the set of data. Of the remaining 35 colonies sampled, close examination of workers’ genotypes revealed 3 or fewer alleles at all four loci in 13 colonies, indicating they were headed by a single queen, and more than three alleles at at least one locus in 22 colonies, consistent with the occurrence of multiple reproductive queens. For the 22 multiple-queen colonies, maximum-likelihood methods detected a number of matrilines per colony ranging from 2 to 8. The spatial distribution of the colonies differed significantly from a random Poisson distribution (Chi-square test: χ^2^ = 3286.12, *df* = 26, *p* < 0.001). The coefficient of dispersion *CD* was 0.71, indicating that colonies were more evenly distributed.

### Aggressiveness

Across the 523 pairwise encounter tests, the intensity of aggression ranged from tolerance (*i*.*e*. score = 0) to fierce fights (*i*.*e*. score = 4). Workers showed no aggressive behaviour when confronted with nestmates (mean aggression score ± SD: 1.19 ± 0.17; min-max: 1–1.33; median = 1.33), whatever their social origin (monogyne: 1.17 ± 0.19, median = 1.17, *N* = 4 pairs of colonies; polygyne: 1.21 ± 0.17, median = 1.33, 8 pairs) (Mann-Whitney *U* test, *U* = 14, *p* = 0.808). Aggression scores from these experiments were therefore pooled for comparisons with encounters between non-nestmates. In contrast, 63% of interactions between workers from different colonies resulted in aggressive responses (mean ± SD = 2.95 ± 1.11; min-max: 0.40–4; median = 3.40). Individuals often engaged in fights with biting and pulling, which could result in the death of one antagonist. The level of aggressiveness was significantly higher during encounters between non-nestmates than between nestmates (Mann-Whitney *U* test, *U* = 516.5, *p* < 0.001). Encounters between two minors or two majors yielded similar behavioural responses, confirming that workers from both castes did not behave differently when confronted with non-nestmates (111 tests on 13 pairs of colonies; minors: mean ± SD = 2.87 ± 1.42, median = 3.80; soldiers: 2.92 ± 1.22, median = 3.80; related-samples Wilcoxon’s signed ranks test, *W* = 13.5, *p* = 0.932).

### Aggressiveness vs. social structure and colony genetic diversity

**Fig 2:**
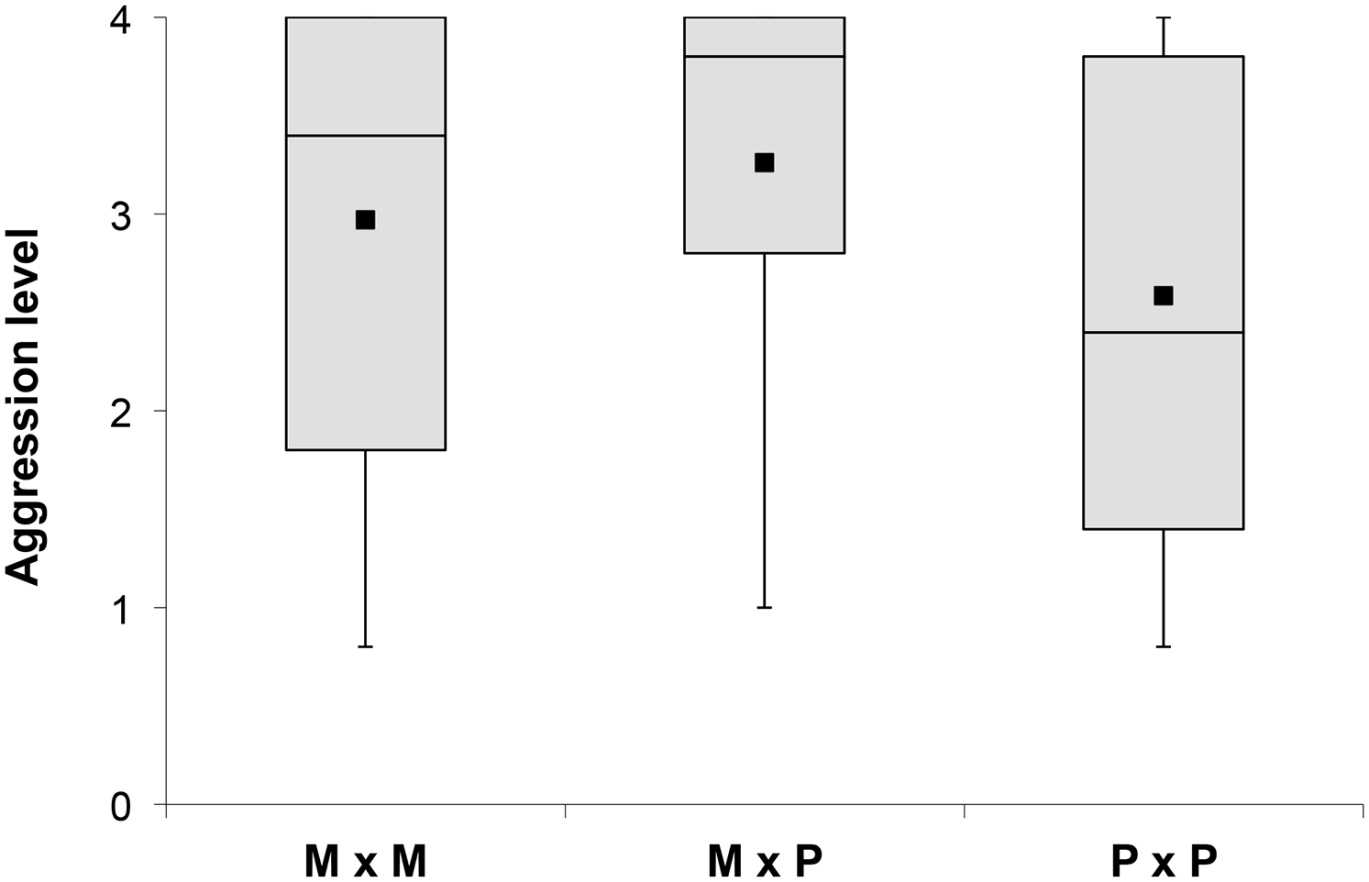
Aggressiveness between workers from monogyne colonies (M x M), from polygyne colonies (P x P) and from monogyne and polygyne colonies (M x P). The closed square indicates the mean of the highest score per replicate, the black bar corresponds to the median, the box is the interquartile range (IQR) and the whiskers represent the lowest datum still within 1.5*IQR of the lower quartile, and the highest datum still within 1.5*IQR of the upper quartile (S2 and S3 Tables).

Regarding non-nestmate encounters, the mean level of aggressiveness was significantly higher between workers from monogyne colonies (M x M) (mean ± SD = 2.97 ± 1.07, median = 3.4, *N* = 59 pairs) than between workers from polygyne colonies (P x P) (2.58 ± 1.18, median = 2.4, *N* = 212) (*p* = 0.049, Dunn's multiple comparison test following significant Kruskal-Wallis test) (Fig 2). Aggression scores during encounters between individuals from monogyne and polygyne colonies (M x P) (3.26 ± 0.94, median = 3.8, *N* = 240) were also significantly higher than scores between workers originating from polygyne colonies (*p* < 0.001), but not higher than scores between workers from monogyne colonies (M x M) (*p* = 0.313) (Fig 2). Finally, confrontation of each colony against all the others showed that the average aggression level was significantly higher for workers from the monogyne colonies (3.15 ± 0.47; median = 3.20) than for workers from the polygyne colonies (2.83 ± 0.36; median = 2.81) (unpaired *t*-test, *t*_30_, *p* = 0.035). Thus, encounters between non-nestmates involving workers from monogyne colonies were always more aggressive.

**Fig 3:**
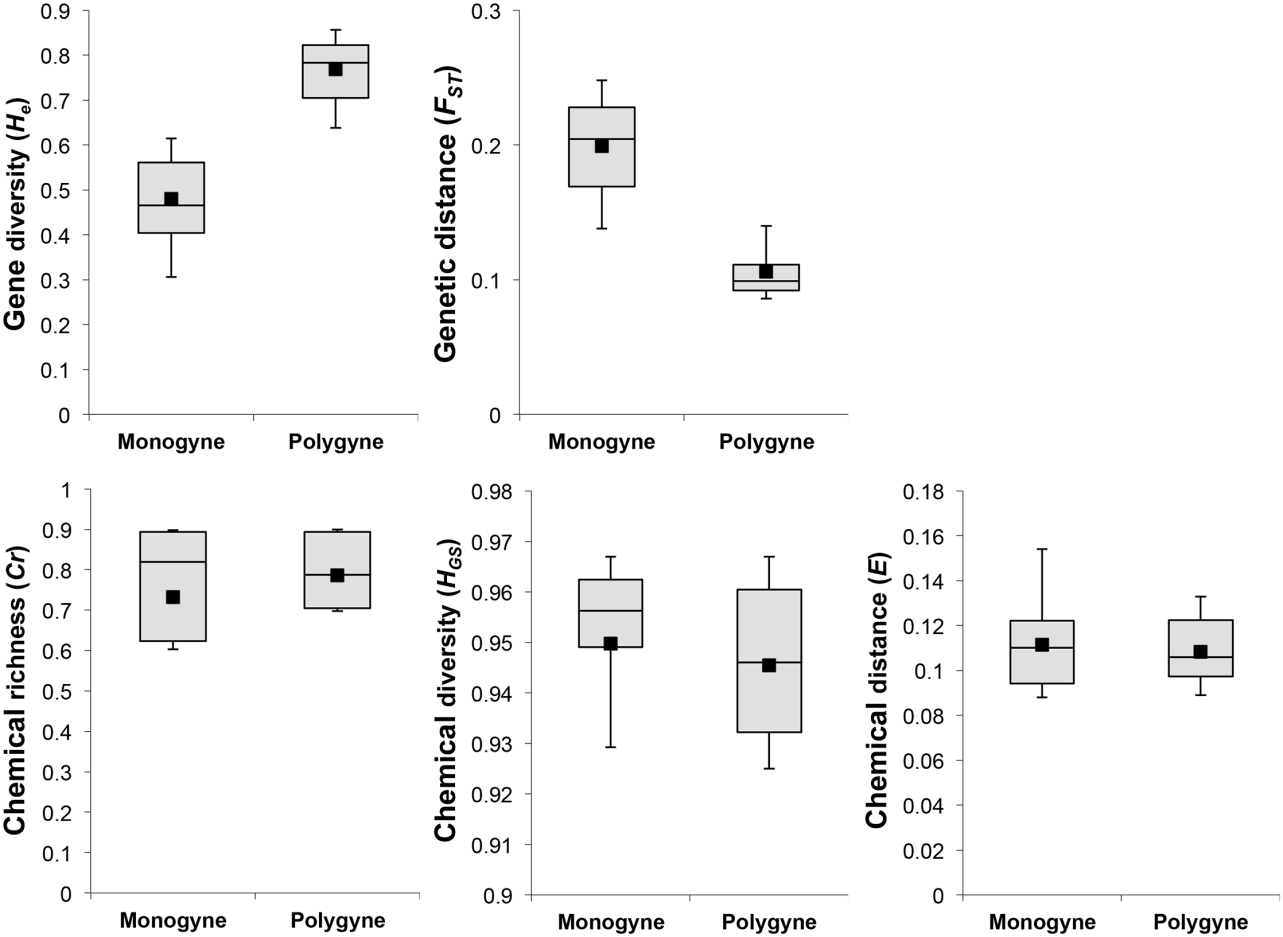
Genetic (gene diversity *He* and pairwise distances *F*_*ST*_; top) and chemical (chemical richness *Cr*, Gini-Simpson index *H*_*GS*_ and the Euclidian distances *E*; bottom) differences between monogyne and polygyne colonies. See Fig 2 for the legend of the box-plot diagram (S3 and S4 Tables).

As expected, multiple-queen colonies were genetically more diverse than single-queen colonies (gene diversity: 0.77 ± 0.07 and 0.48 ± 0.09, respectively; Mann-Whitney *U* test: *U* = 286, *p* < 0.001; Fig 3). Aggressiveness of workers was negatively associated with the genetic diversity within colonies (Spearman rank correlation: *r*_*S*_ = -0.38, *p* = 0.031); the higher the within-colony genetic diversity, the lower the workers’ aggressive behaviour.

### Aggressiveness vs. genetic distances

The genetic distance between colonies was found a good predictor of aggressive behaviour. Mean genetic differentiation between colonies ranged from 0.01 to 0.45. Monogyne colonies were on average significantly more genetically distant from other colonies than polygyne ones (0.20 ± 0.03 and 0.11 ± 0.02, respectively; Mann-Whitney *U* test: *U* = 1, *p* < 0.001; Fig 3). Aggressive behaviour increased with the genetic distance between colonies (Mantel test: *r* = 0.224, *p* = 0.009), with ants from unrelated pairs of nests being more aggressive than those from related nests. This suggests that the level of intraspecific aggression is determined by genetic differences between the protagonists.

Consistent with colony reproduction by budding under polygyny, we found a significant positive correlation between the spatial distance and the genetic distance of colonies (Mantel test: *r* = 0.305, *p* = 0.001). Such an association between the spatial and genetic distances also occurred for monogyne colonies (Mantel test: *r* = 0.303, *p* = 0.012). This indicates that neighbouring nests tend to be more closely genetically related than distant nests in both social structures. A series of Mantel tests in which distant and neighbouring colonies are encoded according to an increasing threshold (*i*.*e*., neighbouring colonies are those below 1 m apart, 1.5 m, 2 m, 2.5 m… up to 40 m apart) showed that up to a radius of 6.0 m around a focal colony, the workers were significantly more related than beyond this value (Mantel tests, *p* < 0.033 for distances below 6.0 m, otherwise *p* > 0.058).

### Aggressiveness vs. spatial distance between colonies

We tested whether the spatial distance between colonies (ranging from 0.77 m to 41.24 m) influenced nestmate recognition. A series of Mantel tests (see above) concluded that up to a radius of 5.0 m, the responses were significantly less aggressive than beyond this value (Mantel tests, *p* < 0.049 for distance below 5.0 m, otherwise *p* > 0.240).

When considering colonies according to their social origin, aggressiveness was positively associated with spatial distance for polygyne colonies (Mantel tests: polygyne colonies: *r* = 0.217, *p* = 0.015), but not for monogyne colonies (*r* = -0.134, *p* = 0.193), suggesting that for polygyne colonies, workers from neighbouring nests behaved less aggressively than workers from distant nests.

### Aggressiveness vs. chemical diversity and distances

The cuticular profile of *P*. *pallidula* was characterized by 61 different hydrocarbons, ranging in size from C_25_ to C_36_. Analyses revealed the presence of lipids including alkanes (26%), mono-methylalkanes (27%), di-methylalkanes (12%), tri-methylalkanes (4%), and alkenes (23%). Identification of peaks and their relative proportion (mean percentage and standard deviation) are listed in Table 1.

**Table 1:** Compounds and their mean percentages (± SD) over all colonies, in monogyne and polygyne colonies. Comparisons between monogyne and polygyne colonies were assessed using Mann–Whitney test; after applying Bonferroni correction, the significance level is set at 0.0008 (*i*.*e*., *α* = 0.05/61 peaks) (S4 Table).

	All colonies	Monogyne	Polygyne	MW *U*-test
	mean ± SD	mean ± SD	mean ± SD	*p*
**Linear alkanes**				
n-C_25_	1.56 ± 2.07	2.04 ± 2.82	1.26 ± 1.43	0.016
n-C_26_	0.52 ± 0.59	0.62 ± 0.38	0.45 ± 0.69	0.024
n-C_27_	2.04 ± 1.38	2.55 ± 1.74	1.72 ± 1.02	0.050
n-C_28_	1.82 ± 0.69	2.08 ± 0.89	1.65 ± 0.48	0.076
n-C_29_	3.63 ± 1.36	3.93 ± 1.42	3.44 ± 1.32	0.624
n-C_30_	3.96 ± 2.09	4.44 ± 2.06	3.67 ± 2.11	0.193
n-C_31_	4.12 ± 1.50	4.71 ± 2.03	3.76 ± 0.94	0.205
n-C_32_	5.23 ± 1.39	5.83 ± 1.64	4.87 ± 1.10	0.082
n-C_33_	0.02 ± 0.07	0.03 ± 0.08	0.01 ± 0.06	0.624
n-C_34_	2.79 ± 0.99	2.86 ± 1.24	2.75 ± 0.84	0.082
n-C_36_	0.21 ± 0.36	0.23 ± 0.40	0.20 ± 0.34	0.600
**Linear alkenes**				
C_26:1_	0.21 ± 0.22	0.22 ± 0.22	0.20 ± 0.23	0.420
C_27:1_	0.45 ± 0.51	0.25 ± 0.29	0.58 ± 0.58	0.042
C_29:1_	3.59 ± 1.95	2.80 ± 1.61	4.09 ± 2.02	0.032
C_29:1_	0.15 ± 0.19	0.10 ± 0.15	0.18 ± 0.21	0.148
C_31:1_	13.49 ± 5.16	11.68 ± 5.17	14.61 ± 4.95	0.046
C_33:1_	0.55 ± 0.25	0.60 ± 0.26	0.52 ± 0.24	0.261
C_33:1_	4.97 ± 2.38	3.99 ± 2.13	5.57 ± 2.37	0.020
**Monomethyls**				
7-Methyl C_25_	0.44 ± 0.89	0.31 ± 0.33	0.52 ± 1.11	0.889
5-Methyl C_25_	0.47 ± 0.37	0.60 ± 0.39	0.38 ± 0.33	0.035
10-Methyl C_26_	0.65 ± 0.68	0.96 ± 0.72	0.47 ± 0.60	0.018
6-Methyl C_26_	0.61 ± 0.53	0.71 ± 0.55	0.55 ± 0.51	0.138
4-Methyl C_26_	0.23 ± 0.38	0.12 ± 0.15	0.29 ± 0.46	0.261
9- + 11- + 13- Methyl C_27_	1.40 ± 0.90	1.78 ± 1.27	1.16 ± 0.47	0.050
7-Methyl C_27_	0.03 ± 0.07	0.03 ± 0.09	0.02 ± 0.05	0.972
5-Methyl C_27_	0.74 ± 0.58	0.71 ± 0.47	0.76 ± 0.64	0.576
3-Methyl C_25_	1.21 ± 0.47	1.35 ± 0.61	1.12 ± 0.34	0.218
12- + 13- + 14- Methyl C_28_	0.47 ± 0.58	0.62 ± 0.92	0.38 ± 0.15	0.675
4-Methyl C_28_	0.32 ± 0.30	0.27 ± 0.21	0.35 ± 0.35	0.484
11- + 13- + 15- Methyl C_29_	1.23 ± 0.42	1.13 ± 0.49	1.28 ± 0.37	0.046
7-Methyl C_29_	0.63 ± 0.25	0.67 ± 0.26	0.60 ± 0.25	0.506
5-Methyl C_29_	0.34 ± 0.23	0.31 ± 0.27	0.35 ± 0.21	0.701
3-Methyl C_29_	2.84 ± 0.94	2.93 ± 1.34	2.78 ± 0.60	1
13- + 14- + 15- Methyl C_30_	1.02 ± 0.58	0.93 ± 0.59	1.07 ± 0.58	0.807
4-Methyl C_30_	0.25 ± 0.26	0.30 ± 0.27	0.23 ± 0.25	0.462
11- + 13- + 15- Methyl C_31_	3.42 ± 0.96	2.73 ± 0.97	3.84 ± 0.69	<0.001*
7-Methyl C_31_	0.47 ± 0.28	0.34 ± 0.26	0.54 ± 0.27	0.007
3-Methyl C_31_	3.89 ± 1.75	4.26 ± 2.76	3.66 ± 0.56	0.600
8- + 9- + 10- + 11- + 12- Methyl C_32_	4.65 ± 1.28	4.76 ± 1.58	4.59 ± 1.10	0.104
15- + 17- Methyl C_33_	0.51 ± 0.33	0.38 ± 0.41	0.59 ± 0.25	0.046
10- + 11- + 12- + 13- Methyl C_34_	0.98 ± 0.93	1.03 ± 0.83	0.95 ± 1.00	0.441
4-Methyl C_34_	0.09 ± 0.24	0.05 ± 0.17	0.12 ± 0.28	0.972
7-Methyl C_35_	0.04 ± 0.22	0.01 ± 0.00	0.07 ± 0.29	0.649
**Dimethyls**				
9,11- + 9,13- + 11,13- +11,15-Dimethyl C_29_	0.21 ± 0.23	0.25 ± 0.29	0.19 ± 0.20	0.861
7,11-Dimethyl C_29_	0.35 ± 0.18	0.35 ± 0.22	0.35 ± 0.16	0.484
3,15-Dimethyl C_29_	1.34 ± 0.72	1.56 ± 1.07	1.21 ± 0.35	0.129
11,15- + 13,15-Dimethyl C_31_	4.65 ± 1.32	4.68 ± 1.57	4.63 ± 1.19	0.807
7,15-Dimethyl C_31_	2.84 ± 1.55	3.19 ± 2.49	2.62 ± 0.36	0.944
5,1- + 5,13- +5,15-Dimethyl C_31_	1.11 ± 0.34	1.13 ± 0.40	1.09 ± 0.30	0.246
7,13-Dimethyl C_33_	0.81 ± 0.67	0.63 ± 0.59	0.91 ± 0.71	0.834
5,13-Dimethyl C_33_	0.14 ± 0.21	0.16 ± 0.24	0.12 ± 0.20	0.753
3,x-Dimethyl C_33_	0.56 ± 0.32	0.42 ± 0.36	0.64 ± 0.26	0.292
5,x-Dimethyl C_35_	0.25 ± 0.29	0.27 ± 0.29	0.24 ± 0.29	0.675
**Trimethyls**				
5,10,12-Trimethyl C_31_	4.43 ± 1.52	4.41 ± 1.86	4.44 ± 1.31	0.462
**Non-identified compounds**				
Peak #10	0.15 ± 0.21	0.17 ± 0.19	0.15 ± 0.22	0.889
Peak #33	0.14 ± 0.15	0.12 ± 0.14	0.15 ± 0.15	0.400
Peak #46	1.14 ± 0.83	0.94 ± 0.86	1.26 ± 0.80	0.552
Peak #50	2.03 ± 0.91	1.92 ± 0.95	2.10 ± 0.91	0.972
Peak #51	0.90 ± 1.18	0.65 ± 1.13	1.05 ± 1.21	0.529
Peak #54	0.28 ± 0.37	0.31 ± 0.37	0.26 ± 0.38	0.780
Peak #55	2.47 ± 0.84	2.63 ± 1.17	2.36 ± 0.56	0.292

Note: The linear alkenes listed twice differ in retention times due to different double bond positions.

A principal component analysis of all cuticular profiles reduced the dimensionality of the data from 61 variables to 13 principal component (PC) factors accounting for 86.86% of the overall variance (only factors with eigenvalues greater than 1 were retained). The chemical profiles of monogyne and polygyne colonies were not differentiated through the discriminant analysis on the 13 PCs: only 58.8% of the colonies were correctly classified according to their social structure (monogyne or polygyne).

We did not find significant difference (after Bonferroni correction) between monogyne and polygyne colonies in relative peak areas (Table 1). Accordingly, the chemical diversity within colonies did not differ between the two social forms (*H*_*GS*_: monogyne colonies: mean ± SD = 0.95 ± 0.02; polygyne colonies: 0.95 ± 0.01; Mann-Whitney *U* test: *U* = 102, *p* = 0.232; *Cr*: monogyne colonies: mean ± SD = 0.73 ± 0.21; polygyne colonies: 0.78 ± 0.02; Mann-Whitney *U* test: *U* = 146, *p* = 0.753; Fig 3) and it was not associated with the genetic diversity within colonies (*H*_*GS*_: *r*_*S*_ = -0.12, *p* = 0.500; *Cr*: *r*_*S*_ = 0.21, *p* = 0.238). Likewise, the distribution of chemical distances *E* was similar across categories of breeding systems (monogyne colonies: 0.11 ± 0.02; polygyne colonies: 0.11 ± 0.01; Mann-Whitney *U* test: *U* = 121, *p* = 0.868; Fig 3). Moreover, we found no association between the chemical distance and the genetic distance between colonies (Mantel test: *r* = 0.115, *p* = 0.108, type II error: ß = 0.515), or between the chemical distance and the aggression level between colonies (Mantel test: *r* = 0.100, *p* = 0.065, ß = 0.557). Thus, the level of intraspecific aggression between colony pairs was not correlated with the similarity of their cuticular lipid profiles.

Chemical distance was also not correlated with spatial distance, neither in polygyne nor in monogyne colonies (Mantel tests: all colonies: *r* = 0.104, *p* = 0.084, ß = 0.661; monogyne colonies: *r* = 0.224, *p* = 0.056, ß = 0.527; polygyne colonies: *r* = 0.032, *p* = 0.314).

Since nestmate recognition may be mediated by few cuticular compounds, notably methyl-branched alkanes [88] or alkenes [89], we repeated statistical analyses separately for each of these classes of cuticular hydrocarbons. Similar results were obtained for all analyses, except three: for unsaturated aliphatic hydrocarbons, there was a positive and significant association between the chemical richness and the genetic diversity (*Cr*_*alkenes*_: *r*_*S*_ = 0.359, *p* = 0.037); for branched alkanes, chemical distances differed between monogyne and polygyne colonies (monogyne colonies: 0.13 ± 0.10; polygyne colonies: 0.10 ± 0.02; Mann-Whitney *U* test: *U* = 69, *p* = 0.016) and were significantly positively associated with aggressiveness (Mantel test: *r* = 0.192, *p* = 0.017).

## Discussion

This study shows that variation in aggressive behaviour toward foreign conspecifics is associated with the level of genetic difference in the ant *P*. *pallidula*. Both the social structure and the genetic distance between colonies are significant predictors of aggression. Workers from monogyne colonies are more aggressive than workers from polygyne colonies. Aggressiveness decreases as genetic diversity within colonies with multiple queens increases. Furthermore, the level of aggression increases with the genetic distance between colonies. The influence of a genetic component on recognition cues and aggressive behaviour has been previously documented in a number of ant species. For example, it has been shown that workers from monogyne colonies are more aggressive toward foreign conspecifics than those from polygyne colonies (*e*.*g*., *Solenopsis invicta*, [57, 90, 91]; *Myrmica rubra*, [38]) and aggressiveness increases as the genetic distance between colonies gets larger (*e*.*g*., *Formica polyctena*, [92]; *Formica pratensis*, [93]; *Crematogaster scutellaris*, [94]). In contrast, such a genetic contribution in discrimination of non-colony members is less apparent, or even absent, in other species where neither the social structure (*Rhytidoponera confusa*, [95]; *Leptothorax ambiguus*, [96]; *Pheidole xerophylla*, [60]; *Formica selysi*, [61]; *Formica fusca*, [63]) nor the genetic distance between colonies (*Pheidole xerophylla*, [60]; *Plagiolepis pygmaea*, [16]; *Acromyrmex lobicornis*, [97]; *F*. *exsecta*, [98]) were found to affect the level of aggression against intruders.

Our data also show that workers behave less aggressively toward individuals coming from nests located within a radius of about 5 m than toward individuals from more distant nests. Similar results were reported in two other *Pheidole* species, *P*. *tucsonica* and *P*. *gilvescens*, where neighbours (*i*.*e*., workers from colonies less than 2.6 m away) are treated less aggressively than workers from more distant nests [99]. This pattern of aggression is consistent with a “dear-enemy” phenomenon, which predicts that individuals are more aggressive toward strangers than neighbours [100]. In *P*. *tucsonica* and *P*. *gilvescens*, the “dear-enemy” phenomenon is mediated by recognition learning [99]: workers habituate to cues provided by individuals from colonies that they regularly encounter (neighbours) and become less aggressive toward them than toward ants with unknown cues (strangers). In an elegant set of experiments, these authors showed that workers from different colonies exposed repeatedly to each other become less aggressive than pairs of workers that were never exposed. The mechanism of habituation would then become predominant for recognition and more significant than other discrimination cues like genetic and environmental ones [99]. One may not completely exclude the possibility of a “dear-enemy” phenomenon based on habituation in *P*. *pallidula*, since this hypothesis has not been explicitly tested. However, the use of genetically based recognition cues is a more parsimonious explanation to account for the lower level of aggression toward neighbours found in this species. In our study population, the genetic distance between pairs of colonies is positively correlated with their spatial distance; this relationship holds for polygyne colonies, as well as for monogyne colonies. Thus, workers from a focal colony are more closely related to neighbours than to individuals from more distant nests. Given that workers’ aggressive behaviour increases with the genetic distance between colonies, the low level of aggression toward neighbours can merely result from their higher relatedness. Consistent with this explanation, partial Mantel tests show that the aggression level is positively associated with the genetic distances between colonies (*r* = 0.224, *p* = 0.009) when controlling for geographic distances, but not with geographic distances between colonies (*r* = -0.013, *p* = 0.447) when controlling for genetic distances.

The pattern of genetic isolation-by-distance uncovered in the monogyne social form of *P*. *pallidula* is somewhat surprising. Previous studies showed that dispersion of colonies in this species can proceed in two ways [41, 65, 101]: (1) long-range nuptial flights and independent colony foundation, and (2) budding, whereby queens mate close to their natal nest and disperse on foot with workers to initiate new colonies nearby. In ants, polygyny is often associated with colony reproduction by budding, which results in populations being genetically structured, whereas monogyny is linked with independent colony foundation and no population structuration (but see [102]). The occurrence of a pattern of genetic isolation-by-distance in the monogyne form of *P*. *pallidula* suggests that a proportion of monogyne colonies may arise by budding from polygyne colonies.

Our chemical analyses did not allow us to show a link between variation in the overall CHC profile and the level of aggression between colonies. We found no difference in the chemical composition, relative peak areas and within-colony chemical diversity between monogyne and polygyne colonies. In addition, we detected no relationship between (*i*) the workers’ aggressive behaviour and the chemical diversity of colonies, (*ii*) the level of aggressiveness and the chemical distance between colonies, (*iii*) the chemical and the genetic distances between colonies, or (*iv*) the genetic diversity of colonies and their chemical richness. Similar results were obtained when considering unsaturated aliphatic hydrocarbons (alkenes) only, with the exception of a positive correlation between genetic diversity and chemical richness of colonies. Although our study was based on a reasonable sample size, we cannot rule out the likelihood that it lacked sufficient power to detect significant effects (post hoc power analyses indeed showed that there were nearly equal chances of incorrectly accepting or rejecting the null hypothesis). Interestingly enough, when considering methyl-branched alkanes only, we found that chemical distances (*i*) differed between monogyne and polygyne colonies, and (*ii*) were significantly associated with aggressiveness. This is consistent with other studies showing that methyl-branched alkanes are key recognition cues in some ant species [29, 88, 103]. Clearly, further studies are needed to confirm these results, notably to elucidate the role of methyl-branched alkanes in nestmate recognition.

Over the last decades, empirical studies have largely supported that cuticular hydrocarbons are responsible for encoding the nestmate discrimination system in ants [28, 29, 88, 89, 104–108]. However, the association between variation in cuticular hydrocarbon profiles, chemical and/or genetic distances, and aggressive behaviour in ants remains somewhat ambiguous. Some studies showed that the level of aggression is positively correlated with the chemical distance between pairs of colonies (*e*.*g*., *Myrmica rubra*, [38]; *Formica exsecta*, [98]), whereas others found no such association (*e*.*g*., *Pheidole megacephala*, [69]). In the same vein, a positive relationship between the chemical distance and the genetic distance between colonies was reported in some species (*M*. *rubra*, [38]; *Cataglyphis niger*, [109]) but not in others (*Crematogaster pygmaea*, [110]; *C*. *scutellaris*, [94]). Comparison of the whole cuticular profile between colonies, though frequently performed, may be too rough an approach, since the CHCs extracted from workers are not all involved in nestmate discrimination [88, 98, 103, 107, 111]. In *P*. *pallidula*, the absence of a relationship between the level of aggression toward foreign conspecifics and the chemical profile of colonies might stem from a lack of knowledge about the cuticular compounds directly involved in nestmate discrimination. Identifying these key compounds would lead to an understanding of how aggressiveness of workers is linked to chemical recognition cues. In this respect, the role of methyl-branched alkanes in nestmate recognition in *P*. *pallidula* certainly merits further study.

## Supporting Information

S1 Table Matrix of geographic distances between colonies (m). (CSV)

S2 Table Matrix of agressiveness between colonies. (CSV)

S3 Table Microsatellite data. (CSV)

S4 Table Areas of peaks identified as cuticular lipids. (CSV)
